# The effect of metaverse on L2 vocabulary learning, retention, student engagement, presence, and community feeling

**DOI:** 10.1186/s40359-024-01549-4

**Published:** 2024-02-02

**Authors:** Ferdi Çelik, Meltem Huri Baturay

**Affiliations:** 1 Ondokuz Mayıs University, Samsun, Türkiye; 2 Konya Food and Agriculture University, Konya, Türkiye

**Keywords:** Metaverse, Language education, Teaching, Engagement, Learning

## Abstract

This study investigates the multifaceted impacts of Metaverse Based Language Teaching on high school L2 vocabulary learning and retention, engagement, community feeling, and diverse dimensions of presence, namely social, cognitive, teaching, and overall presence. Adopting a quasi-experimental design, the study provides an in-depth controlled trial. The participants in this study consist of 86 (43 male, 43 female) high school English language learners in Türkiye, who are assigned to two groups. The control group uses traditional instructional technologies while the experimental group engages with metaverse technology. The lessons are based on social constructivist theory for both groups. Pre- and post-tests are employed to quantitatively assess vocabulary learning and retention and reveal substantial improvements in both areas. A series of carefully selected psychometric scales capture core aspects of engagement, community feeling, and presence. The findings reveal increased levels of engagement and a sense of presence, and connectedness within the language learning community, which confirms the efficacy of the MBLT. However, the improvement in social presence does not reach statistical significance. Cumulatively, the findings of this research transcend mere quantifiable improvements in vocabulary learning and retention. They reveal a holistic transformation of the language learning experience by emphasizing the significance of social interactions and digital embodiment in high school second language education through metaverse. The research highlights the metaverse’s potential in shaping the future of language education, reimagining traditional paradigms, and proposing a new era of immersive, interactive, and transformative pedagogy.

**Supplementary Information:**

The online version contains supplementary material available at 10.1186/s40359-024-01549-4.

## Introduction

If we were to search for the prompt “photo of a classroom in the 1950s” in a search engine and compare the results with today’s classrooms, we might be surprised not by their stark differences but by their striking similarities. Then, one may wonder why the classrooms remain almost the same while almost everything changes. In an era defined by a growing need for innovative educational approaches, the metaverse, an immersive environment, has the potential to revolutionize traditional pedagogical practices. As the boundaries between the physical and digital worlds continue to blur in other sectors, educators and researchers are increasingly exploring the transformative power of the metaverse in shaping the future of education [1]. Metaverse can offer unique opportunities for students that they may be unable to access in their real lives due to economic, social, and practical limitations. It is often challenging or impractical to go beyond the concrete walls of our classrooms during the lesson physically. However, we can help learners explore the *affordances* (learning opportunities) outside those walls virtually. As in explorative learning, the metaverse provides students with authentic experiences [2], and it may encourage the development of communicative competence through social interactions with others [3]. Rather than writing on a chalkboard or a smartboard, teachers can design fully personalized smart learning environments tailored to students’ needs and the lesson’s objectives in the metaverse.

The integration of technology, particularly in language teaching, has witnessed remarkable advancements that open up new possibilities for engaging and interactive learning experiences [4–10]. The metaverse concept, which encompasses interconnected virtual worlds and shared digital spaces, adds a new dimension to the potential applications of virtual environments in language teaching. Metaverse offers students the opportunity to engage in exploratory language learning activities within computer-generated, interactive, and often immersive settings [11–16]. For example, in the metaverse, students can interact with other English-speaking individuals from different parts of the world, explore their cultures, engage in activities, and learn from each other. The immersive experiences within the metaverse may replicate specific scenarios that allow learners to practice foreign/second language skills [4–6] and expand their vocabulary repertoire in relevant and meaningful contexts [9]. Such an immersive metaverse space can be designed to enhance second language (L2) vocabulary learning, which is a fundamental aspect of second/foreign language learning [17].

Nevertheless, in traditional language teaching, L2 learners often face slower progress due to limited exposure to the target language (TL) and have lower language practice opportunities compared to those immersed in an L2 environment [18–20]. This may hinder vocabulary learning and retention because students learn more vocabulary in an environment where higher levels of involvement load [10, 21, 22] and exposure frequency [19, 23] is offered. Metaverse can be designed as a solution to address this issue. Therefore, motivated by the United Nation’s Sustainable Development Goal 4: Quality Education [9, 24], this paper aims to provide a novel approach to ELT by using the metaverse for L2 pedagogy and investigates the effect of the metaverse on English language learners’ vocabulary learning and retention, classroom community feeling, engagement, and their social, cognitive, teaching, and overall presence levels.

### Metaverse-based language teaching

Linguistic environment plays a crucial role in L2 learning and acquisition [25]. Metaverse platforms are highly immersive since they can integrate virtual reality (VR), augmented reality (AR), mixed reality (MR), extended reality (XR), and other digital technologies that enhance the feeling of reality [26]. It might be tempting to ask why we need a *virtual* reality when there is *reality*. The answer is simple: the reality we desire or need may often be unavailable or inaccessible. For instance, hypothetically, an L2 teacher would like to teach new vocabulary about aquatic life in the ocean. Could they bring the ocean into the L2 classroom? With metaverse, they can virtually teach about marine life by interacting with the learners under a virtual ocean. Thus, Metaverse-Based Language Teaching (MBLT) has emerged as a promising technological-pedagogical approach in the field of English Language Teaching (ELT) parallel to the growth of AR/VR/MR/XR and AI technologies and the increasing popularity of virtual environments and the growth of web 3.0 platforms. The metaverse itself is not a novel concept [27], and it was coined in 1992 by the novelist Neal Stephenson in his science fiction book Snow Crash. Metaverse refers to a virtual world or a collective virtual shared space that is created [28] by the convergence of various digital technologies and media, such as audio, video, images, animations, 3D objects and interactive elements such as slideshows, where users can interact with each other [14, 29] and with the environment in a simulated 3D space [30]. Metaverse is a persistent virtual space that can be accessed and experienced by learners through the use of avatars [31], which are the digital representations (online or digital self) of the learners. These avatars are believed to represent Gen Z learners well in a digital environment [32–34]. While the use of avatars in the metaverse provides several benefits, the utilization of avatars has also raised concerns, including but not limited to matters about privacy, the safeguarding of intellectual property rights, and the potential for personal harm [35], especially in platforms in which users are provided with high degrees of freedom and anonymity [28]. For that reason, taking specific precautions might benefit such as setting metaverse space as private, agreeing on metaverse classroom rules with the students, and getting supervisor control over the students (i.e., ability to summon students near your avatar, or limiting the mobility of an avatar).

Metaverse allows for various forms of social [30], economic [36], and cultural activities, which provide several opportunities for human interaction [12], communication [37], and creativity [3]. Metaverse is also practical, affordable [38], and environmentally friendly, especially compared to the physical world. This is because the cost of accessing metaverse is as low as having a smartphone, tablet, or computer connected to the internet [39]. However, more immersive experiences require VR headsets that are relatively expensive. Furthermore, it can be accessed worldwide, eliminating location constraints. Web 3.0-powered metaverse platforms may integrate various foundational AI technologies to provide immersive and interactive experiences [40], such as sensors for visual perception, natural language processing for speech recognition and generating human-like language and language understanding, machine learning algorithms for personalization, and virtual assistants for user interaction and guidance. This demands a newer definition of metaverse than previously offered in the literature [14]. In the context of this study, the metaverse is an interconnected realm of immersive virtual worlds empowered by advanced digital technologies, which fosters user interaction within a simulated 3D environment and facilitates seamless integration with diverse Web 3.0 platforms across various devices by serving a multitude of purposes.

As the metaverse can host various forms of digital content with multiple technologies, it can be embedded into ELT educational materials to create affordances [41] for learners. It is indicated in the academic literature that such virtual environments increase learners’ motivation, self-confidence [42, 43], achievement [44], communication, and collaboration [45], teamwork skill and self-directed learning [46] and engagement [9]. The metaverse has found integration in diverse educational contexts, including but not limited to pediatric dentistry [47], language learning [48], educational equity [12], aircraft maintenance education [49], information technology education [50], and the teaching of history [51].

Given that the MBLT is a contemporary concept, the theoretical foundation still needs to be developed within Computer-Assisted Language Learning (CALL) research and practice. CALL often implements a range of theoretical frameworks such as Interactionist Second Language Acquisition (ISLA), Social Constructivism, and Sociocultural Theory in various instructed ELT settings [52]. ISLA, credited to Long [25], emphasizes the negotiation of meaning in interactions between learners and speakers of the target language. On the other hand, social constructivism asserts that learners construct knowledge via social interactions, emphasizing the role of social and cultural factors in learning [53]. Based on Vygotsky’s work, sociocultural theory stresses the importance of cultural values in shaping learning [54]. Using these theories, the English language learning process can be enhanced through the real-time multisensory social interactions offered in MBLT [55].

Therefore, ISLA’s emphasis on negotiated meaning finds resonance in MBLT’s facilitation of real-time, multisensory social interactions, which contributes to an enriched language learning experience. Social Constructivism manifests in MBLT’s collaborative learning approach within the metaverse, which fosters knowledge construction through interaction. Sociocultural Theory is integrated into MBLT by recognizing the culture in language learning, and enhancing language learning with diverse and culturally relevant content. Together, these theoretical foundations form a comprehensive framework, which elevate MBLT as a dynamic techno-pedagogical approach in English language education.

### The definition of cognition within MBLT

Recent research in cognitive science informs CALL research and practice that cognition and body may not be isolated from each other [56–58], and action may facilitate the language learning process [59–61]. As the metaverse allows learners to have a virtual body through avatars [62], they embody their digital selves parallel to their real selves. Within embodied cognition, cognitive processes, such as perception and language, are grounded in bodily experiences and interactions with and within an environment [63]. In other words, our thoughts and actions are not merely determined by the brain but are also influenced by the physical and social context in which we exist. For instance, our interactions with objects within an environment lead to the creation of mental representations concerning their characteristics and potential uses, subsequently influencing our future actions and thoughts regarding those objects. In a similar vein, the metaverse holds the capacity to elevate our embodied experiences, offering fresh avenues for engaging with and delving into virtual environments.

Virtual embodied cognition is the idea that our cognition can be influenced by the experience of being embodied in a virtual environment via an avatar [64–66]. This may play a key role in how the metaverse facilitates learning by providing higher degrees of social presence [37, 67, 68], enjoyment [69] and cognitive abilities [69, 70].

Engaging in an embodied learning environment raises several essential considerations. These include assessing the extent to which learners are truly immersed in the experience, the strength of their sense of community, their awareness of other instructors, and their cognitive engagement with the learning activities. Despite being rooted in extended reality (XR) technologies, the metaverse is still in its early stages of development, and its potential and the various factors that influence learning within this environment are underexplored. At this juncture, it is essential to investigate how students perceive their presence in the learning environment and the emotional aspects associated with their experiences. These issues should be approached not only from a cognitive perspective but also from an affective standpoint.

### Community feeling

Community feeling is a profound and often intangible aspect of human interaction that manifests in a strong emotional bond among classroom members, such as the teacher and the learners. In L2 settings, it refers to the sense of belonging, connection, and shared identity that individuals experience within an L2 classroom [71]. The MBLT presents a promising environment for enabling social interaction, fostering communication, and providing exposure to target language input. Using the metaverse for language learning can include integrating social and cultural elements by creating engaging virtual environments that facilitate culturally appropriate social interactions [53, 54] for language learners. Therefore, it can potentially enhance the classroom community feeling [72–74] by allowing students to interact with each other and their instructor in a virtual space in meaningful ways, which might foster a sense of belonging and collaboration.

### Presence

The existing research highlights the potential impact of presence on the learning process [75–79]. Three key constructs inform us about learning in a community of inquiry [78], grounded in constructivism [11, 80, 81]. Cognitive presence refers to the extent to which learners can construct and confirm their understanding of a subject or topic through collaborative and reflective discussion. Another one, teaching presence refers to the design, facilitation, and direction of instructors that they often provide in virtual or blended learning environments. This presence is not limited to the instructor; it can also involve peer teaching and collaboration, where learners take on teaching roles in group projects or discussions. Finally, social presence refers to the degree to which participants in a virtual interaction or community perceive and experience a sense of connection, sociability, and the presence of others despite the physical separation in online interactions. It is an indicator of a rich and engaging digital learning experience. Nevertheless, some studies have been inconclusive in confirming a direct link between social presence and the learning outcomes [82, 83].

### User engagement

While there have been several studies investigating L2 learner engagement [71, 84, 85], the user engagement comes into play when evaluating the engagement during the process of human-computer interaction. User engagement refers to a dimension of user experience defined by the extent of a user’s cognitive, temporal, affective, and behavioral commitment during interactions with a digital technology [86]. The determinants of user engagement are diverse and depend on the context [87]. Personal factors, including demographics, cognitive abilities, and personality traits, may shape the way individuals engage with digital content [88]. System-related factors, such as usability, design aesthetics, and functionality, may also play a crucial role in attracting and retaining users [89]. Additionally, social factors, such as social presence contribute significantly to user engagement in online environments [90].

### Use of Spatial.io as a tool for MBLT

Over a decade, many scholars have found the positive impacts of the metaverse in learning and teaching, and most of these studies have used similar software such as *Second Life* [43, 64, 91–95] which is an internet-based multimedia platform enabling individuals to craft personalized avatars and engage with fellow users and user-generated content in a collaborative virtual world environment. A meta-review examined 167 empirical studies on 3D virtual learning environments in education and revealed that *Second Life* was one of the most popular platforms, with case studies and quasi-experimental designs being common and language learning extensively studied [16]. However, due to its practical limitations, many educators have been cautious about incorporating *Second Life* into their curricula.

Notably, old-generation metaverse platforms such as *Second Life* lacks cross-platform compatibility, restricting access to those with computers or laptops, which not all students possess or can easily transport to school. Another significant issue with older metaverse platforms is the requirement for users to download software and updates [96], which can disrupt the classroom environment, with some students unprepared to join and others left waiting. Furthermore, some of the old metaverse platforms’ open-ended nature can distract learners, as they may lack clear objectives for learners to complete [97] and may raise concerns about safety due to its unrestricted access [98]. Moreover, old metaverse platforms falls short in their foundational support for new technologies such as spatial computing, AR/VR, blockchain and Web 3.0 platforms although the widespread recognition of the metaverse today can be attributed to the emergence of these advanced technologies. Platforms such as *Second Life* do not allow educators to easily structure the virtual environment for particular teaching contexts and provide them with control over the teaching process. As a cutting-edge metaverse platform, *Spatial* allows learners to create personalized virtual avatars and immersive spaces for live events and social interactions. With *Spatial*, learners can step into the metaverse via their smartphones, tablets, and computers, or for enhanced reality, they can use VR headsets. In it, the students may feel like they are truly transported to a different place that is distinguishable from just browsing a website or watching a video. *Spatial* is practical and adaptable, so that it may be suitable for almost any lesson. Upon critically reviewing the literature on the use of metaverse in language education, we believe modern metaverse platforms like *Spatial* offer much more than conventional metaverse platforms.

*Spatial* empowers learners to individualize their educational journey by creating avatars and immersive environments to foster self-directed and tailored learning. This enables them to align their virtual environment with their personal interests, preferences, and educational objectives. Such personalized learning experiences have the potential to sustain learner engagement and motivation [37]. Spatial might also be helpful for learners to interact with each other and their teacher in a virtual space. The learners can collaborate on tasks, participate in group discussions, and receive feedback on their language skills. This interactive learning can help learners develop their communication skills, e.g., while learning English. Last but not least, *Spatial* can create authentic environments, which can help learners practice real-life scenarios. For example, learners can practice ordering food at a restaurant, buying a ticket at a train station, or negotiating a business deal. These simulations can help language learners build their confidence and fluency in the target language. In the current study, a modern metaverse platform, *Spatial*, is investigated comprehensively in the context of CALL research and practice.

*Spatial* can be especially useful for teaching and learning English. Firstly, it can simulate real-life situations and allow learners to practice their English in a safe, authentic, and supportive environment with a highly immersive and interactive language learning experience. As it allows learners to create customized virtual spaces and privacy settings, only those you let can access the environment, making it safer for pedagogical use. Secondly, *Spatial* may help learners improve their L2 skills by creating vivid memories of shared experiences beyond mere observations of educational videos or listening to pre-written texts, making them active in the learning process. This can also improve learners’ memory retention by eliminating the external stimuli caused by the physical environment [99]. Learners may better remember a conversation with a more knowledgeable English speaker in a virtual environment than watching a video or reading a text, as it is highly immersive.

### Empirical studies informing MBLT

Metaverse offers novel perspectives to the education system by letting learners go beyond the brick-and-mortar walls of the physical schools and engage with new learning opportunities within a virtual environment [100]. Recent research on the integration of metaverse has revealed promising opportunities in the field of L2 teaching and learning, such as increasing teaching efficacy [101], motivation [42], confidence [102], willingness to speak [103], and achievement [37, 104]. Virtual learning environments (VLE) facilitate L2 learning by reducing anxiety [105, 106], decreasing timidity and fear of embarrassment [91], promoting practical collaborative tasks [17] collaboration [45]. Recent studies have also shown that the use of VLEs can improve speaking and listening skills in English [2, 42, 105, 107–110].

### The gap and the present study

The literature reveals significant gaps in MBLT research and practice. These gaps include studies on modern metaverse platforms such as Spatial.io, a lack of exploration of MBLT, an underdeveloped theoretical framework for MBLT, the need for understanding its place under CALL, unaddressed privacy and ethical concerns, and the absence of empirical studies evaluating the effectiveness of MBLT. Addressing these gaps is crucial for advancing our understanding of MBLT. Therefore, in the current study, we propose *Spatial* platform to design an innovative, educational, smart, and interactive MBLT environment. We believe that MBLT can enhance any language skill when effective pedagogical strategies are incorporated. However, in the present study, we commenced by examining the effect of using *Spatial* as an MBLT tool on L2 vocabulary learning and retention, focusing on the following research questions:

1. How does a metaverse-based vocabulary learning environment influence high school L2 students’ vocabulary learning and retention?
2. To what extent does a metaverse-based vocabulary learning environment with a social constructivist approach impact the engagement of high school L2 students?
3. What is the effect of a metaverse-based vocabulary learning environment on the development of community feeling among high school L2 students?
4. In what ways does a metaverse-based vocabulary learning environment with a social constructivist approach influence the social, cognitive, teaching, and overall presence levels of high school L2 students?

## Method

### The research design

The present study employed a quasi-experimental design [111] with one control group and one experimental group. This design was chosen to investigate the effects of using metaverse on high school student’s vocabulary learning and retention, as well as on their cognitive, social, and teaching presence in the environment, students’ sense of classroom community and engagement. Randomly assigning students to metaverse and non-metaverse groups was not feasible because the high school had pre-determined classes in which students were placed. A quasi-experimental design allowed for the study of naturally occurring groups: different classes of high school students. Therefore, all the classes in the high school in grades nine, 10, and 11 included in the study and these classes randomly assigned to control and experimental groups to have equal chance of being selected as a control or experimental group.

### Study context

The present study took place at a private high school in Türkiye, which was managed by a non-profit organization. The high school was part of a campus with two other buildings including kindergarten, primary and secondary school. There were three languages that were taught: English, German, Russian and French. The students were instructed with 10 h of compulsory English weekly while they learned one additional language based on their selection among the other languages two times a week. Each classroom had a smartboard, internet connection and at most 24 desks. There were eight classrooms with two classrooms for each grade level from grade nine to 12. The English language teachers were provided with continuous professional development opportunities including seminars and webinars by the Foreign Languages Directorate of the organization. The students were instructed with National Geographic’s Perspectives Coursebooks during the year in which the study was conducted. The language of instruction was English only in the English course, and the students used their mother tongue in all the other lessons.

### The participants

86 high school students (43 males and 43 females) participated in the study. The students were learning English as a primary and compulsory foreign language. The participants were selected based on purposeful sampling [112]. The inclusion criteria required participants to be enrolled in L2 classes at the high school level. The participants were aged between 14 and 17. The students were divided into control and experimental groups, with 43 students in each group. The groups were matched in terms of their average proficiency in English with, which was determined based on their scores in a test covering listening, reading, writing, and use of English by a standardized test. Their proficiency level ranged between B1-B2. Each group consisted of an equal number of students from grades 9, 10, and 11, which ensures a balanced representation (Table 1).

**Table 1:** Demographics of the participants

Items	f	%
***Gender***		
Male	43	50
Female	43	50
***Age***		
13–15	42	48.84
16–18	44	51.16
***Metaverse Experience***		
No experience	24	27.91
Very little	14	16.28
Little	19	22.09
Moderate	15	17.44
High	9	10.47
Very high	26	5.81
**Total (for each item)**	**86**	**100**

Table 1 illustrates that females and males and age groups were almost proportionate. The students were divided into control and experimental groups, keeping the proportioning almost the same.

### The instruments

The study aimed to examine the effect of using metaverse on vocabulary learning and retention of L2 learners, their cognitive, social, and teaching presence; engagement and sense of classroom community feeling.

#### Vocabulary learning and retention test

A new vocabulary learning test [see Supplementary file 1] was developed by one of the researchers, who was an L2 teacher to be used in the present study. The test items were carefully designed to measure participants’ knowledge and retention of vocabulary specifically related to the lesson materials and aligned with the course’s learning objectives. The test was a five-option multiple-choice test consisting of 20 items. Each item was worth five points, and the maximum score a student could get was 100. After the test was developed, it underwent a rigorous review process by three experienced English language teachers who taught students at the same high school level. This ensures the test’s validity and reliability. The test was administered as a pre-test before the intervention that served as a baseline measurement and the same test was utilized again as a post-test immediately after the completion of the intervention. This pre-and post-test design allowed for an assessment of the impact of the metaverse intervention on participants’ vocabulary learning and retention, and it provided valuable data for the study. 25 days after the last intervention, the same test was applied again to measure retention. In pre-, post-, and retention tests, we randomized the order of questions and the options to enhance the internal validity.

#### The community of inquiry (CoI) framework

The CoI framework [113], was used to measure cognitive, social, and teaching presence in the metaverse environment. The questionnaire assessed learners’ perceptions of the presence concerning quality of these three dimensions in the classroom environment. The questionnaire consisted of 34 Likert-type items rated on a 5-point scale, ranging from 1 (strongly disagree) to 5 (strongly agree). The questionnaire was administered to the participants at the end of the intervention. The internal consistency (Cronbach’s Alpha) resulted in scores of 0.94 for teaching presence, 0.91 for social presence, and 0.95 for cognitive presence [113]. The questionnaire was used as a pre and post-test for both metaverse and non-metaverse groups.

#### Sense of classroom community scale

The scale helps measure the strength and quality of relationships and interactions among students and between them and their instructors. To measure students’ community and connectedness feelings in the metaverse learning environment, we used the Classroom Community Scale developed by Rovai [114]. It was adapted and validated to be used for a virtual learning environment with the intra-class correlation coefficients for the overall scale, as well as for the connectedness and learning sub-dimensions are 0.939, 0.935, and 0.944, respectively; and internal consistency scores were 0.87, 0.86 and 0.85, respectively [74]. The scale comprises 20 items using a five-point Likert-style format, where respondents express their agreement or disagreement to scale items [74]. The scale used as a pre- and post-test for both control and experimental groups.

#### User engagement scale

The scale was designed by [87] to assess how engaged and involved users are when interacting with technology, such as websites, apps, or digital content. The User Engagement Scale [87] was chosen as it was a reliable and up-to-date tool that was designed to measure user engagement focusing on evaluating how engaging a technological tool was for a user. The scale has four key dimensions: Focused Attention, Perceived Usability, Aesthetic Appeal, and Reward Factor, which collectively contributed to the overall User Engagement score.

We used the short form of the scale, which consisted of 12 items and included five dimensions: focused attention (α = 0.92), perceived usability (α = 0.92), aesthetic appeal (α = 0.90), and reward factor (α = 0.87). The scale utilized a 5-point Likert-type response format from strongly disagree to strongly agree. The scale was used as a post-test for the experimental group and was not applied to the control group as the aim of it was to understand user engagement within the metaverse environment.

### Procedure

A metaverse environment was designed in Spatial.io by one of the researchers [115]. The design process involved user-friendly drag-and-drop controls; no coding knowledge was required. Participants could navigate in the metaverse environment by using the WASD buttons on the keyboard to move their avatars and the mouse’s left-click button to interact with objects. The researchers utilized the Isle Gallery template, which was selected based on the aim of the lesson and the preferred pedagogical procedures.

**Fig. 1:**
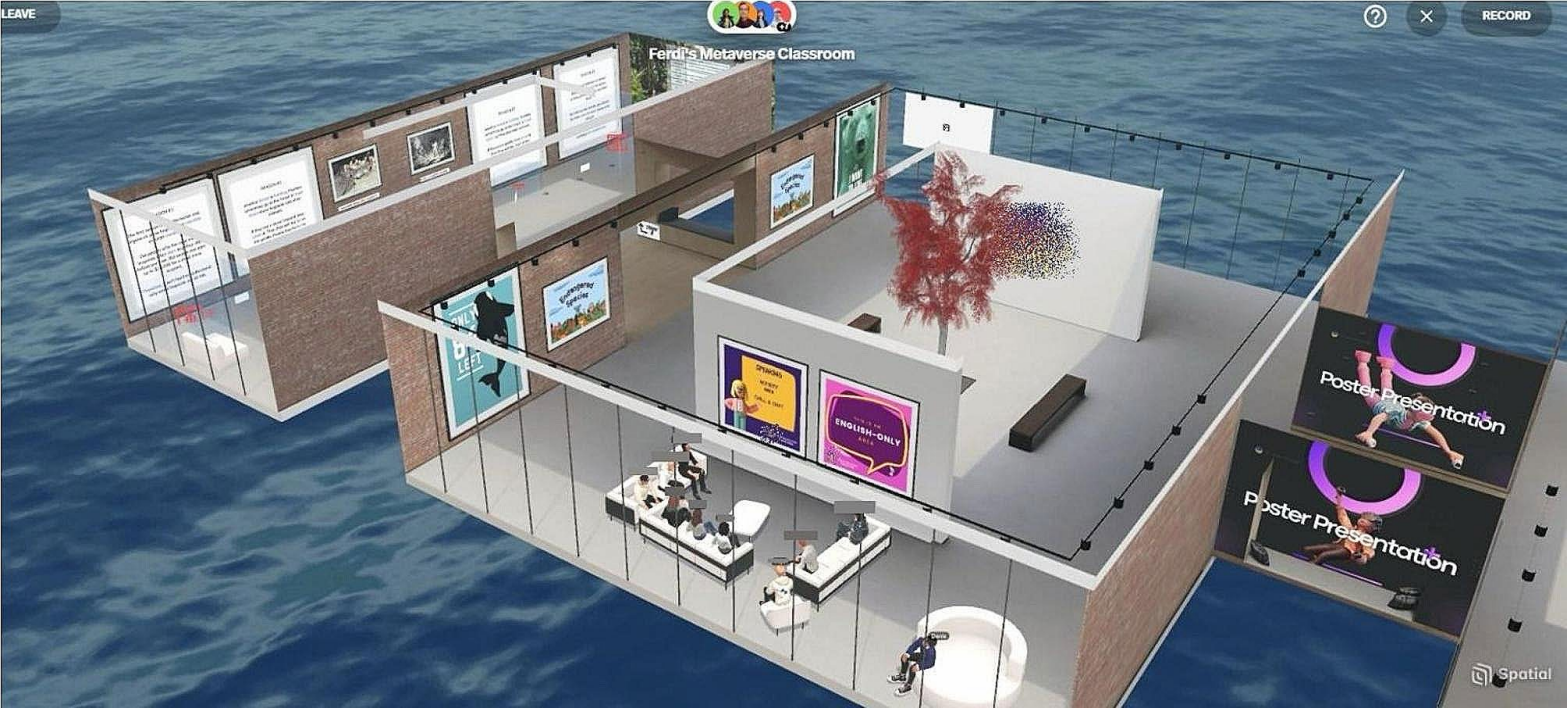
Overview of the designed Metaverse

The metaverse space included the presentation of target vocabulary-related 3D objects in flashcards, animated pictures (GIFs), static pictures, and texts (Fig. 1). Next, an MBLT lesson plan was prepared to ground it in the social constructivist theory of SLA [see Supplementary file 2].

The lesson plan included various activities, such as a jigsaw reading activity, group work, and group discussions, to promote vocabulary learning and engagement. Collaborative learning is a prominent feature of *social constructivism*. The lesson plan encourages students to work in pairs during various stages of the lesson, such as when they gather information about their assigned animals and engage in discussions about raising awareness. Collaborative learning enables students to engage in meaningful dialogue, share perspectives, and *co-construct knowledge*. By working together, students can build on each other’s ideas, challenge assumptions, and better understand the subject matter. The interactive elements facilitate active engagement and exploration. These tools allow students to seek information, ask questions, and interact with the content, the teacher, and their peers. This aligns with the social constructivist belief that knowledge is actively constructed through dialogue, inquiry, and hands-on exploration. Questioning and discussion play a pivotal role. Students are encouraged to generate their questions about the endangered animals and engage in discussions with their peers.

### Data collection process

After obtaining informed consent of the participants’ parents, verbal assent from the participants, permission to conduct the study from the school administration and its headquarters and ethics committee approval from the university, the data collection process was commenced. The instruments were delivered to the participants in a carefully organized sequence. The sequence included pre-intervention, during-intervention, and post-intervention phases and are summarized as follows:

#### Pre-intervention

Before the intervention, the researchers obtained ethical approval from the Ethics Committee. Informed consent forms were prepared and distributed to the parents/legal guardians of the participating students. During this phase, the participants’ demographic information, including age, gender, and grade level, was collected. Additionally, the participants’ proficiency in English was assessed using a standardized test. The test assessed participants’ listening, reading, writing skills, and use of English. The test results were used to ensure that the control and experimental groups had similar English proficiency levels.

Participants were then assigned to the control or experimental groups based on their class enrollment. The assignment was done to ensure an equal number of students from grades 9, 10, and 11 in both MBLT and traditional language teaching groups. While the control group students were exposed to regular English language instruction, the experimental group students participated in the vocabulary learning activities in the tailored MBLT environment. Following these, the vocabulary learning pre-test, classroom community scale pre-test and CoI framework pre-test were implemented to measure the vocabulary levels of the participants prior to the MBLT intervention. The structure of the delivery times is illustrated in Table 2.

**Table 2:** Instrument delivery times

The Instrument	Pre-Intervention	During Intervention	Post- Intervention	After Intervention
Demographics Survey	X			
Vocabulary Learning Scale	X		X	X
Classroom Community Scale	X		X	
User Engagement Scale*		X		
Community of Inquiry Framework	X		X	

*This scale was only delivered to the experimental group

#### During intervention

Several steps applied during the intervention. The treatment phase involved the implementation of two different lesson plans for the control and experimental groups.

**Fig. 2:**
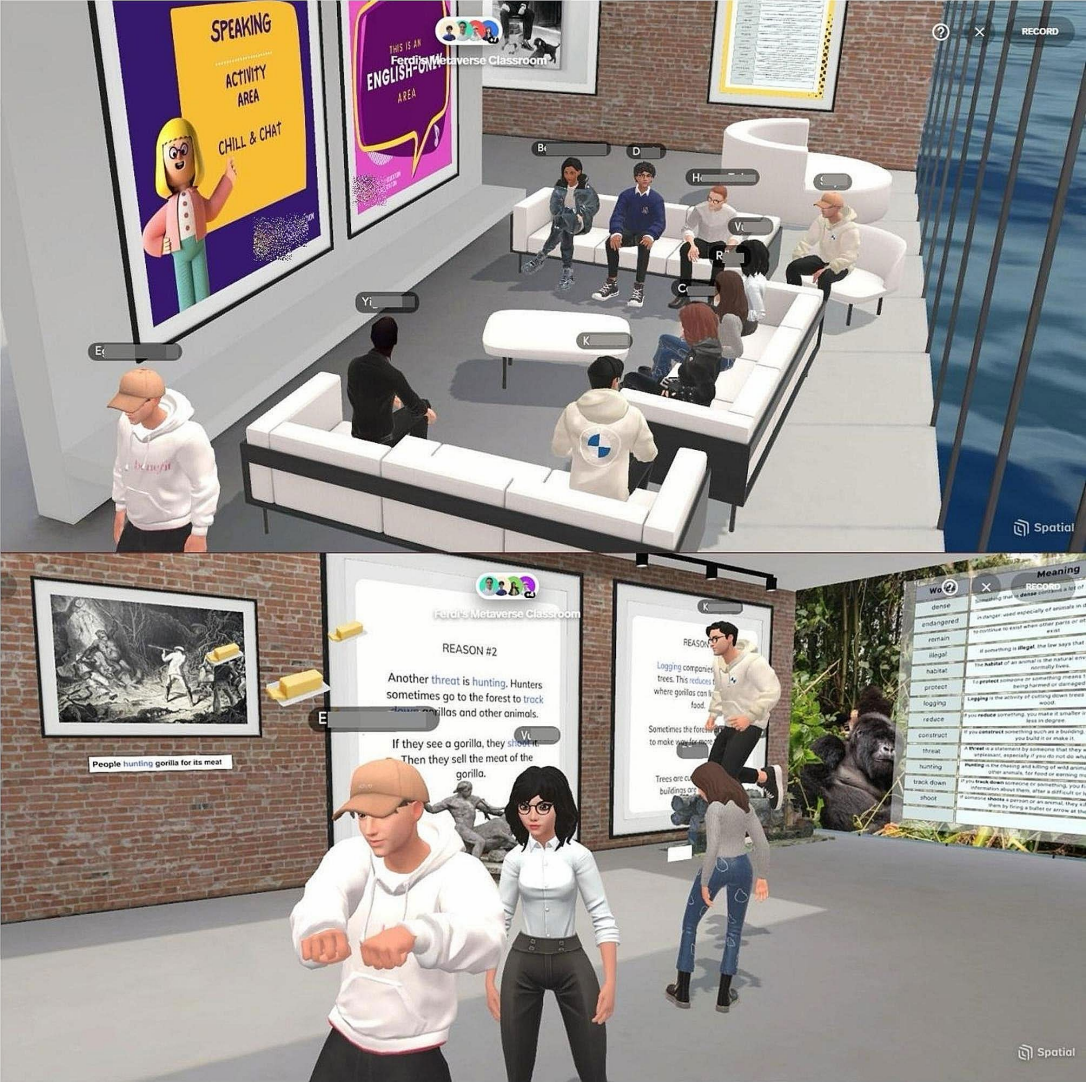
Students having vocabulary class in Metaverse

*Experimental Group*: The experimental group engaged in vocabulary learning activities in the tailored metaverse environment created in Spatial.io (Fig. 2). A lesson plan was designed by the researchers based on activities grounded in social constructivism [see Supplementary file 2]. The lesson plan incorporated the interactive features of the metaverse, allowing students to explore 3D objects, view animated pictures, access static pictures, and interact with textual information on the topic. The students navigated in the metaverse environment using avatars, and the researchers taught English within the virtual space based on the lesson plan (Fig. 2). The lesson for the experimental group took 80 min.

*Control Group*: The control group received the same instruction with the same learning objectives and similar steps but utilizing traditional technologies rather than metaverse. This included the use of MS PowerPoint presentations and digital flashcards. The teacher followed the pre-determined lesson plan, covering the vocabulary learning objectives using MS PowerPoint slides and the classroom presentation software of the coursebook. The lesson of the control group lasted for 80 min.

Both groups received instruction over several sessions, following the same duration and frequency of practice. The lesson plans were designed to address specific vocabulary learning objectives and promoted student engagement and interaction. The user engagement scale was delivered to experimental group participants during this period.

#### Post-intervention

After completing the intervention, the post-test scales for measuring cognitive, social, and teaching presence and sense of classroom community were delivered to both groups of participants to measure the outcomes of the intervention. 25 days after the participants had taken the post-test, the vocabulary learning post-test was delivered to the participants in both groups as a retention test. After the data collection process ended, the control group was also received the same metaverse based instruction to have the same educational opportunities.

### Data analysis

The data were structured in an MS Excel file for data analysis. The structured data were analyzed using the Statistical Package for the Social Sciences (SPSS) 26 software in Windows 11.

#### Vocabulary learning and retention

We compared L2 learners’ achievement mean gain between pre-and post-test. Shapiro-Wilk normality tests indicated non-normal data distributions for both the pre-test and post-test scores (pre-test: *p* = 0.049; post-test: *p* < 0.001). Consequently, non-parametric analysis was chosen as the appropriate method. We applied the Wilcoxon signed-rank test to determine changes within the Control and Experimental groups. The Mann-Whitney U test was then used to compare these groups’ achievement mean gains.

#### User engagement

As the control group did not use the metaverse, we only applied the user engagement scale to the experimental group. To analyze user engagement, we assessed various dimensions: Focused Attention, Perceived Usability, Aesthetic Appeal, and Reward Factor by conducting descriptive statistics. These dimensions were combined to calculate an overall engagement score. The data analysis sought to determine the levels of user engagement within the Metaverse environment, particularly in terms of each dimension’s mean scores.

#### Social presence

To examine the impact of MBLT vocabulary instruction on students’ social presence, we conducted a Shapiro-Wilk normality test. The test results indicated a non-normal data distribution in the control group (*p* = 0.015). Thus, a Mann-Whitney U test was chosen to compare social presence between the control and experimental groups.

#### Teaching, cognitive, and overall presence

We assessed the teaching, cognitive, and overall presence within the MBLT vocabulary lesson. Shapiro-Wilk normality tests confirmed that the data for these dimensions were normally distributed (*p* > 0.05). Therefore, independent sample t-tests were conducted to determine the differences in teaching, cognitive, and overall presence between the control and experimental groups.

### Ethical considerations

#### Ethical approval

for the study was obtained from a university’s Social Research Ethics Committee. Informed consent forms were provided to the participant’s parents or legal guardians, explaining the study’s purpose, procedures, potential risks, and benefits. The study ensured participant confidentiality and anonymity throughout the data collection and analysis. All the procedures employed in this study were conducted according to the Declaration of Helsinki.

## Results

### Vocabulary learning and retention

To determine the effect of using Metaverse on students’ vocabulary learning, we analyzed pre and post-test data. Shapiro Wilk normality test was run, and it was seen that the data were not distributed normally (*p* = 0.049 for the pre-test and *p* < 0.001 for the post-test). Thus, we conducted a non-parametric analysis. The results are illustrated in Table 3.

**Table 3:** Wilcoxon signed-rank test for the Vocabulary Learning test

Post-test–pre-test	N	Mean Rank	Sum of ranks	Z	*p*
*Control*	43				
Negative ranks	4	14.25	57	−3.745	< 0.001*
Positive ranks	26	15.69	408		
Ties	13				
Total	43				
*Experimental*	43				
Negative ranks	3	16.33	49	−4.694	< 0.001*
Positive ranks	35	19.77	692		
Ties	5				
Total	43				

*p < 0.05

As seen in Table 3, the Control group’s post-test scores (M = 51.40, SD = 19.34) exhibited a statistically significant increase compared to their pre-test counterparts (M = 41.86, Z = -3.745, *p* < 0.05). Similarly, the post-test scores demonstrated a significant increase in vocabulary learning compared to pre-test scores (Z = -4.694, *p* < 0.05) in the experimental group. The mean gain between pre- and post-tests for the Control Group (M = 35.35) was lower than the Experimental Group (M = 51.65). Thus, we further compared whether the difference between the two groups was significant (Table 4).

**Table 4:** Comparison of vocabulary learning test mean gains between control and experimental group

	N	M	Sum of ranks	U	Asymp. Sig. (2-tailed)
Control	43	35.35	1520	574	0.002*
Experimental	43	51.65	2221		
Total	86				

*p < 0.05

The results provided that the difference was significant. Cohen’s d was calculated as 0.981, indicating a large effect size for the metaverse intervention. Next, we ran a normality test to evaluate the impact of a metaverse-based vocabulary lesson on vocabulary retention. The test indicated that data were not normally distributed (*p* = > 0.05). A Mann-Whitney U test was employed to compare the control and experimental groups regarding retention (Table 5).

**Table 5:** Comparison of Experimental and Control Groups in terms of Vocabulary Retention

	N	M	Sum of ranks	U	Asymp. Sig. (2-tailed)
Experimental	43	48.86	2101	694	0.008*
Control	43	38.14	1640		
Total	86				

*p < 0.05

The results of the vocabulary retention tests for the experimental group (M = 48.86) and the control group (M = 38.14), demonstrated a statistically significant difference in the means in favor of the control group with a two-tailed *p*-value of 0.008 (*p* < 0.05).

The study also assessed the impact of using a metaverse-based vocabulary lesson on students’ vocabulary retention. Due to the data being not normally distributed, a Mann-Whitney U test was conducted to compare the vocabulary retention scores.

The results revealed a statistically significant difference in vocabulary retention between the experimental group (Mann-Whitney U = 694, M = 48.86) and the control group (M = 38.14), U = 1640, with a two-tailed *p*-value of 0.008. The significance level, denoted by “p” value, was set at 0.05. Therefore, the observed *p*-value (*p* < 0.05) indicated a significant difference between the two groups regarding vocabulary retention.

### Classroom community

After calculating the mean difference between pre-test and post-test for experimental and control groups, the level of improvement in the community feeling, with respect to connectedness and learning factors was measured. To assess the normality of our data distribution, we first conducted the Shapiro-Wilk normality test. The results indicated that all dimensions of the scale and the overall scale data were normally distributed (*p* > 0.05). This suggested that the data met the assumption of normality, required to conduct parametric statistical tests. Thus, we conducted independent sample t-tests for each dimension: connectedness, learning, and community feeling. Table 6 illustrates the findings.

**Table 6:** Independent Samples T-test for Community Feeling Scale Data

		F	Sig.	t	df	Sig. (2-tailed)	Mean Difference	Std. Error Difference	95% Confidence Interval of the Difference	
									Lower	Upper
Connectedness	Equal variances assumed	0.648	0.423	3.460	84	0.001	0.830	0.240	0.353	1.307
	Equal variances not assumed			3.460	82.174	0.001	0.830	0.240	0.353	1.308
Learning	Equal variances assumed	0.140	0.709	3.827	84	0.000	0.907	0.237	0.436	1.378
	Equal variances not assumed			3.827	83.992	0.000	0.907	0.237	0.436	1.378
Total	Equal variances assumed	0.102	0.751	3.704	84	0.000	0.869	0.234	0.402	1.335
	Equal variances not assumed			3.704	83.361	0.000	0.869	0.234	0.402	1.335

#### Connectedness

In the context of connectedness, the analysis indicated a significant difference between the two groups. When assuming equal variances, the t-test yielded a t(84) = 3.460 with a *p*-value of 0.001. The connectedness experienced by the experimental group was significantly higher than the control group. Cohen’s d was calculated as 0.746, indicating a medium effect size.

#### Feeling of learning

Regarding the feeling of learning, the analysis revealed a significant difference between the two groups, again favoring the experimental group. When assuming equal variances, the t-test yielded a value of t(84) = 3.827 with a *p*-value of > 0.001, indicating a highly significant difference. Cohen’s d was 0.825 that indicated a large effect size.

#### Community feeling

With respect to community feeling, the analysis results indicated a significant difference between the two groups. When equal variances were assumed, the t-test yielded a value of t(84) = 3.704 with a *p*-value of < 0.001, signifying a significant divergence. The mean difference of 0.869 and a 95% confidence interval ranging from 0.402 to 1.335 imply that the experimental group experienced a notably higher sense of community within the learning environment than the control group. Cohen’s d was found as 0.799 with a medium effect size.

Overall, the results indicated that using the intervention had a statistically significant positive impact on connectedness, learning, and community feeling on experimental group students when compared to control group that was instructed without metaverse.

### User engagement

**Fig. 3:**
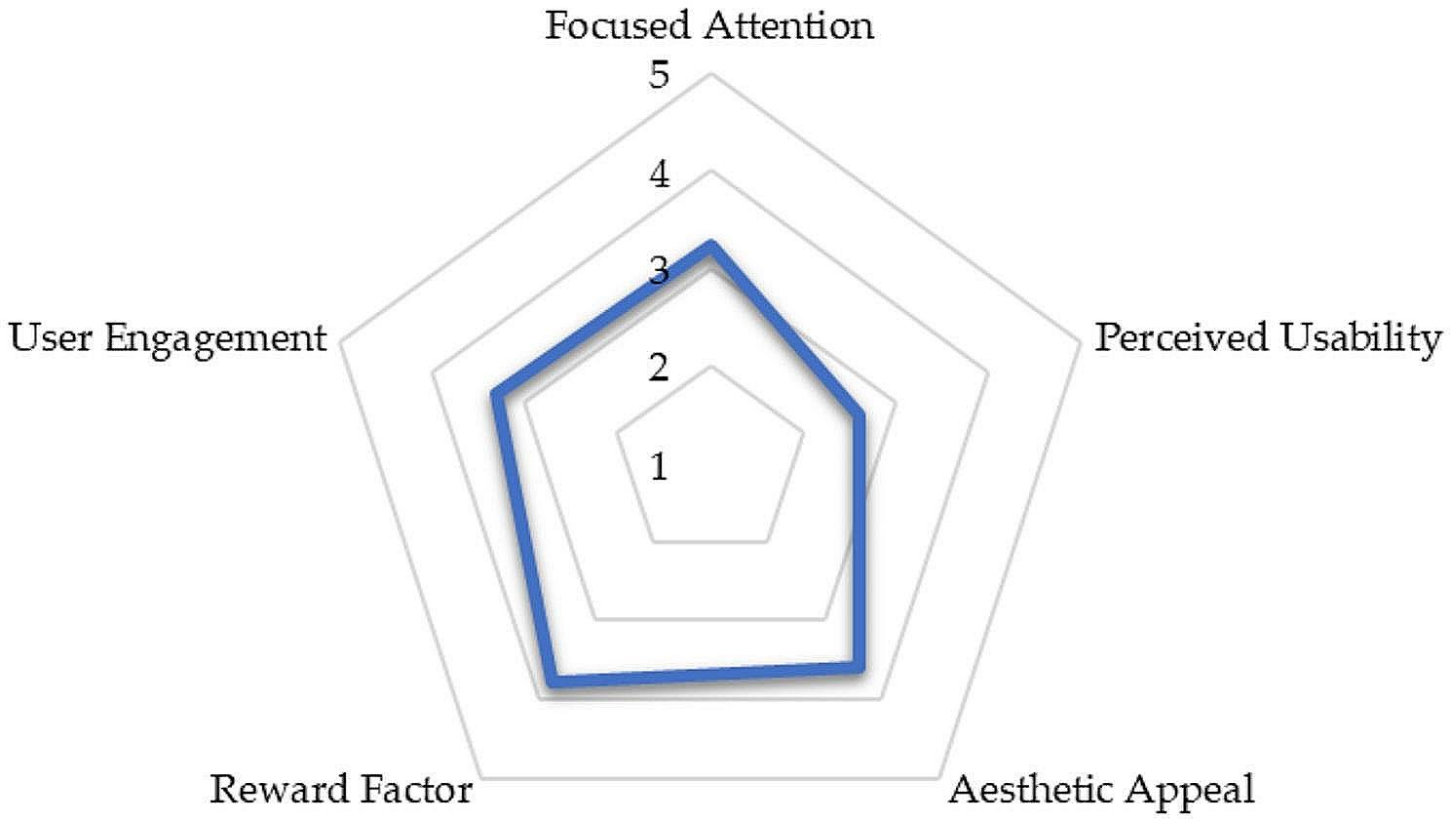
User Engagement Rates

To analyze user engagement, we analyzed it through various five dimensions: Focused Attention, Perceived Usability, Aesthetic Appeal, and Reward Factor by conducting descriptive statistics. Figure 3 illustrates the findings:

The data revealed that participants exhibited a moderate level of Focused Attention (M = 3.23, SD = 1.08) (Fig. 3). The mean score for the dimension of Perceived Usability was relatively lower (M = 2.61, SD = 0.97). The participants found the Aesthetic Appeal of the metaverse environment to be relatively high (M = 3.60, SD = 0.918). The Reward Factor dimension, on the other hand, yielded a mean score of 3.77. Overall, learners found metaverse engaging (M = 3.30, SD = 0.547).

### Presence

The data for social presence were not normally distributed based on the Shapiro Wilk normality test (*p* = 0.015 for the control group). Therefore, we employed the Whitney U test (Table 7).

**Table 7:** Mann Whitney U Test Results for Social Presence

	N	M	Sum of ranks	U	Asymp. Sig. (2-tailed)
Experimental	43	47.58	2046	749	0.129
Control	43	39.42	1695		
Total	86				

As Table 7 illustrates, the mean for the experimental group was M = 47.58, while the mean for the control group was M = 39.42. This suggests that, on average, participants in the experimental group had a mean score in social presence than those in the control group. However, the Mann-Whitney U test yielded a *p*-value of 0.129, indicating that this difference is not statistically significant at the 0.05 significance level.

We also examined teaching, cognitive, and overall presence within the metaverse setting. Normality tests confirmed that the data for these dimensions were normally distributed (*p* > 0.05), enabling us to proceed with independent sample t-tests, as outlined in Table 8.

**Table 8:** Comparison of presence among experimental and control group

		Levene’s Test for Equality of Variances		t-test for Equality of Means						
		F	Sig.	t	df	Sig. (2-tailed)	Mean Difference	Std. Error Difference	95% Confidence Interval of the Difference	
									Lower	Upper
Teaching	Equal variances assumed	0.184	0.669	2.829	84	0.006	0.726	0.257	0.216	1.237
	Equal variances not assumed			2.829	83.941	0.006	0.726	0.257	0.216	1.237
Cognitive	Equal variances assumed	3.934	0.051	2.294	84	0.024	0.541	0.236	0.072	1.009
	Equal variances not assumed			2.294	77.989	0.024	0.541	0.236	0.071	1.010
TOTAL	Equal variances assumed	0.328	0.568	2.349	84	0.021	0.555	0.236	0.085	1.026
	Equal variances not assumed			2.349	83.147	0.021	0.555	0.236	0.085	1.026

For teaching presence, Levene’s test indicated equal variances (F = 0.184, *p* = 0.669). The t-test revealed a statistically significant mean difference (t = 2.829, df = 84, *p* = 0.006). This signifies that the experimental group (M = 0.726) demonstrated higher teaching presence than the control group, with a 95% confidence interval ranging from 0.216 to 1.237 (Table 8). Cohen’s d was also calculated (Cohen’s d = 0.609) and resulted in a medium effect size. For cognitive presence, the Levene’s test suggested unequal variances (F = 3.934, *p* = 0.051). The t-test, assuming unequal variances, showed a significant difference in means (t = 2.294, df = 77.989, *p* = 0.024). This underscores that the experimental group (M = 0.541) displayed more fabulous cognitive presence than the control group, with a 95% confidence interval from 0.072 to 1.009. Cohen’s d for this comparison resulted in (Cohen’s d = 0.494) a small effect size. Regarding overall presence, equal variances were assumed (F = 0.328, *p* = 0.568). The t-test yielded a statistically significant mean difference (t = 2.349, df = 83.147, *p* = 0.021). This implies that the experimental group (M = 0.555) exhibited a higher overall presence than the control group, with a 95% confidence interval spanning from 0.085 to 1.026. Cohen’s d for overall presence comparison was calculated and concluded with a medium effect size (Cohen’s d = 0.505).

## Discussion

The results of this research study provided valuable insights into the impact of utilizing the Metaverse as an educational tool in enhancing students’ vocabulary learning, fostering a sense of classroom community and presence, and promoting user engagement. The pre and posttest data analysis revealed a statistically significant improvement in vocabulary learning among students in both the Control and Experimental groups. The experimental group, which utilized the Metaverse, exhibited more vocabulary learning improvement than the Control group, suggesting that the immersive and interactive nature of the Metaverse contributes positively to vocabulary learning outcomes; thus, its potential as a powerful tool for vocabulary learning. Furthermore, our findings indicated that metaverse-based vocabulary improved students’ vocabulary retention. These findings were consistent with prior research demonstrating the effectiveness of immersive environments in educational settings [11, 12, 37, 43, 64, 91–94, 104, 116–119].

The study also investigated the impact of the Metaverse on the sense of community among L2 students. Our data demonstrated a significant difference in community feeling between the Metaverse-instructed and traditional groups. Students in the Metaverse-instructed group reported higher levels of connectedness, learning satisfaction, and community feeling. This suggests that the Metaverse can provide a sense of belonging and connectedness among students, fostering a supportive learning environment. The findings align with the notion that virtual environments can facilitate social interactions [3, 12–15, 120] and a sense of community in blended education settings [32].

Assessing user engagement was crucial for understanding how students interacted with the metaverse as an educational platform. Previous studies in the literature highlight that the metaverse is an engaging tool for learning [49, 121]. In the present study, the results indicated that participants exhibited moderate levels of focused attention, indicating that the Metaverse activities captured students’ attention. The high rating for the Reward Factor dimension suggested that gamification elements in the Metaverse positively influenced the engagement levels of the students. However, the relatively lower score for Perceived Usability indicates that participants encountered some challenges in the Metaverse environment. To enhance the overall user experience, educators and developers should address usability issues. By giving clear instructions, they may guide students in the Metaverse platform.

The Aesthetic Appeal dimension received positive ratings, indicating that the multimodal aspects of the Metaverse made it a pleasing learning environment. This finding underscores the importance of creating engaging and aesthetically appealing educational virtual environments. The assessment of teaching, cognitive, and overall presence within the metaverse-based vocabulary class indicated that the environment positively impacted students’ feeling of presence. This could be attributed to the interactive and immersive nature of the metaverse, which enables more instructor-student interactions [4–8, 10–15]. Similarly, it improved students’ cognitive presence suggesting that the lesson may facilitate deeper thinking processes and knowledge construction among students. It may encourage students to explore and use their cognitive skills more effectively. Lastly, the metaverse-based vocabulary class showed a substantial advantage in terms of overall presence which may be attributed to the combined enhancements in teaching and cognitive presence.

Examining social presence within the metaverse-based vocabulary lesson yielded intriguing yet statistically inconclusive results. On average, participants in the experimental group appeared to exhibit higher social presence than their counterparts in the control group, indicating the potential for enhanced connection and engagement. However, the difference was not statistically significant, which supported the previous findings in the literature [82, 83]. This suggests that metaverse-based vocabulary classes may offer comparable social presence to conventional classes, but not superior. Nevertheless, further in-depth and longitudinal research is required to explore social presence dynamics within virtual learning environments comprehensively. In conclusion, the findings of this study present a nuanced perspective. While social presence did not exhibit statistical significance, teaching, cognitive, and overall presence significantly improved within the metaverse-based vocabulary class. These results underline the potential of metaverse in language teaching and warrant further investigation into the specific issues and best practices within metaverse-based learning environments.

To recap, the findings revealed a substantial positive effect of the metaverse on students’ vocabulary learning, with improvements in vocabulary and retention levels. This underscores the metaverse’s capacity to enhance language learning outcomes, emphasizing the value of interactive and gamified approaches to vocabulary instruction. Furthermore, the study shed light on the MBLT’s potential to foster a strong sense of classroom community among L2 students. This indicates the power of immersive and interactive virtual environments in creating supportive and engaging learning atmospheres, especially significant in remote or online educational settings with the augmentation of connectedness, learning satisfaction, and community feeling. The lesson plan which is grounded in Social Constructivism was effective for vocabulary learning in the metaverse, highlighting the alignment of it with the MBLT. The current study exposed a nuanced landscape when analyzing user engagement within the MBLT. Students displayed focused attention and a sense of reward, indicating that gamification elements in the MBLT successfully captured their interest. However, challenges linked to perceived usability were evident, highlighting the necessity for continual improvements in the usability of virtual learning platforms. The positive evaluation of the Metaverse’s aesthetic appeal further contributed to the overall user experience, enhancing engagement.

### Limitations

Firstly, due to the inherent characteristics of our research, random assignment was impractical, which led our study to adopt a quasi-experimental design. In a typical educational setting, such as the one under investigation, students are typically organized into classes. The challenge arose in randomly selecting students from all classes and assigning them to either the metaverse or non-metaverse groups individually. Consequently, we employed a random assignment approach at the level of the school’s classes rather than at the level of individuals. Secondly, the present study assessed the effect of a short-term intervention. Longitudinal studies could offer more insights into using MBLT as a long term learning approach. Thirdly, participants entered the study with varying levels of metaverse experience. The extent of one’s familiarity with the metaverse may influence their engagement with the study. Next, the metaverse environment was designed by one of the researchers, and alternate designs may yield different outcomes. Further studies with diverse designs are warranted for a comprehensive understanding. Lastly, although our study incorporated a retention test conducted 25 days post-intervention, the relatively short follow-up period restricts our ability to evaluate the prolonged durability of the observed effects. A longitudinal study could offer valuable insights into the sustained impact of metaverse intervention on vocabulary learning. Given these limitations, readers are advised to interpret the study results accordingly.

## Conclusion

The current study has deepened our understanding of how the Metaverse can improve educational outcomes, promote a sense of community, and engage students in innovative and immersive ways. The results of this study have significant implications for the field of foreign and second language education, which demonstrates that the use of the MBLT positively impacts vocabulary learning, fosters a sense of community, and enhances user engagement in the context of ELT. Thus, using the MBLT can make language more engaging, interactive, and community-oriented. Educators and instructional designers are suggested to explore the potential of the metaverse to enhance vocabulary acquisition and promote a sense of connectedness among students. ELT teachers are encouraged to integrate MBLT into their high school syllabus by ensuring alignment with learning objectives and proficiency levels. Customized learning modules within the Metaverse may be designed to cater to the linguistic needs and interests of high school students. Activities within the Metaverse should prioritize cultural and contextual relevance that could make language learning more meaningful for high school students.

To capitalize on these benefits, it is essential to address usability issues and continuously improve the design and functionality of the Metaverse for educational purposes. This study serves as a starting point for further research and development in this exciting field and it hopes to have contributed the way for innovative approaches to L2 teaching and learning. These results offer an outlook for integrating immersive virtual environments in educational settings and call for further exploration of the potential of the Metaverse in improving learning outcomes and the overall educational experience.

### Electronic supplementary material

Below is the link to the electronic supplementary material.

**Supplementary Material 1:** Vocabulary Learning and Retention Test

**Supplementary Material 2:** Metaverse-Based Language Teaching Lesson Plan

## Data Availability

No datasets were generated or analysed during the current study.

## References

[1] Phakamach P, Senarith P, Wachirawongpaisarn S (2022). The metaverse in education: the future of immersive teaching & learning. RICE J Creative Entrepreneurship Manage.

[2] Lan Y-J, Liao C-Y (2018). The effects of 3D immersion on CSL students’ listening comprehension. Innov Lang Learn Teach.

[3] Polys N, Roshan S, Newton E, Narula M, Thai B-T. Designing for social interactions in a virtual art gallery. In: *Proceedings of the 27th International Conference on 3D Web Technology: 2022*; 2022:1–9.

[4] Annamalai N, Uthayakumaran A, Zyoud SH (2023). High school teachers’ perception of AR and VR in English language teaching and learning activities: a developing country perspective. Educ Inform Technol.

[5] Bonner E, Lege R, Frazier E. Teaching CLIL courses entirely in virtual reality: educator experiences. Calico J. 2023;40(1).

[6] Dooly M, Thrasher T, Sadler R. Whoa! Incredible! Language learning experiences in virtual reality. Relc J. 2023:00336882231167610.

[7] Yuan J, Liu Y, Han X, Li A, Zhao L. Educational metaverse: an exploration and practice of VR wisdom teaching model in Chinese Open University English course. Interact Technol Sma. 2023.

[8] Faramarzi S, Dayag JD. Augmented reality and virtual reality: new frontiers in technology-enhanced language learning. Perspectives on enhancing learning experience through digital strategy in higher education. edn.: IGI Global; 2023. pp. 166–89.

[9] Çelik F, Yangın Ersanlı C (2022). The use of augmented reality in a gamified CLIL lesson and students’ achievements and attitudes: a quasi-experimental study. Smart Learn Environ.

[10] Yanagisawa A, Webb S (2022). Involvement load hypothesis plus: creating an improved predictive model of incidental vocabulary learning. Stud Second Lang Acquisition.

[11] Hwang H, Kim H (2023). Automatic analysis of constructional diversity as a predictor of EFL students’ writing proficiency. Appl Linguist.

[12] Zhai X-s, Chu X-y, Chen M, Shen J, Lou F-l. Can Edu-Metaverse reshape virtual teaching community (VTC) to promote educational equity? An exploratory study. IEEE Trans Learn Technol. 2023.

[13] Wang Y, Lee L-H, Braud T, Hui P. Re-shaping Post-COVID-19 teaching and learning: a blueprint of virtual-physical blended classrooms in the metaverse era. In: *2022 IEEE 42nd International Conference on Distributed Computing Systems Workshops (ICDCSW): 2022*: IEEE; 2022:241–247.

[14] Park S-M, Kim Y-G (2022). A metaverse: taxonomy, components, applications, and open challenges. IEEE Access.

[15] Ning H, Wang H, Lin Y, Wang W, Dhelim S, Farha F, Ding J, Daneshmand M. A survey on the metaverse: the state-of-the-art, technologies, applications, and challenges. IEEE Internet Things. 2023.

[16] Reisoğlu I, Topu B, Yılmaz R, Karakuş Yılmaz T, Göktaş Y (2017). 3D virtual learning environments in education: a meta-review. Asia Pac Educ Rev.

[17] Chen JC (2018). The interplay of tasks, strategies and negotiations in Second Life. Comput Assist Lang Learn.

[18] Lee S-M. Factors affecting incidental L2 vocabulary acquisition and retention in a game-enhanced learning environment. ReCALL. 2022:1–16.

[19] Teng MF (2022). Incidental L2 vocabulary learning from viewing captioned videos: effects of learner-related factors. System.

[20] Tsai Y-L, Tsai C-C (2018). Digital game-based second-language vocabulary learning and conditions of research designs: a meta-analysis study. Comput Educ.

[21] Kolaiti P, Raikou P (2017). Does deeper involvement in lexical input processing during reading tasks lead to enhanced incidental vocabulary gain. Stud Engl Lang Teach.

[22] Eckerth J, Tavakoli P (2012). The effects of word exposure frequency and elaboration of word processing on incidental L2 vocabulary acquisition through reading. Lang Teach Res.

[23] Xiaoning C, Feng T (2017). Assessing the effects of word exposure frequency on incidental vocabulary acquisition from reading and listening. Chin J Appl Linguistics.

[24] Lee BX, Kjaerulf F, Turner S, Cohen L, Donnelly PD, Muggah R, Davis R, Realini A, Kieselbach B, MacGregor LS (2016). Transforming our world: implementing the 2030 agenda through sustainable development goal indicators. J Public Health Policy.

[25] Long M. The role of the linguistic environment in second language acquisition. *Handbook of second language acquisition*. 1996.

[26] Ball M. Framework for the metaverse: the metaverse primer: matthewball vc.; 2021.

[27] Novak K (2022). Introducing the Metaverse, again!. TechTrends.

[28] Kye B, Han N, Kim E, Park Y, Jo S. Educational applications of metaverse: possibilities and limitations. J Educational Evaluation Health Professions. 2021;18. 10.3352/jeehp.2021.18.32 PMC8737403 34897242

[29] Jovanović A, Milosavljević A (2022). VoRtex Metaverse platform for gamified collaborative learning. Electronics.

[30] Xu X, Zou G, Chen L, Zhou T. Metaverse space ecological scene design based on multimedia digital technology. *Mobile Information Systems 2022*, 2022.

[31] Khansulivong C, Wicha S, Temdee P. Adaptive of new technology for agriculture online learning by metaverse: a case study in faculty of agriculture, national university of Laos. In: 2022 *Joint International Conference on Digital Arts, Media and Technology with ECTI Northern Section Conference on Electrical, Electronics, Computer and Telecommunications Engineering (ECTI DAMT & NCON): 2022*: IEEE; 2022: 428–432.

[32] Tlili A, Huang R, Shehata B, Liu D, Zhao J, Metwally AHS, Wang H, Denden M, Bozkurt A, Lee L-H (2022). Is metaverse in education a blessing or a curse: a combined content and bibliometric analysis. Smart Learn Environ.

[33] Duan H, Li J, Fan S, Lin Z, Wu X, Cai W. Metaverse for social good: a university campus prototype. In: *Proceedings of the 29th ACM international conference on multimedia*: 2021; 2021:153–161.

[34] Earnshaw R, Sourin A. Case study: shared virtual and augmented environments for creative applications. Res Dev Acad Creative Industries Appl. 2017:49–64.

[35] Cheong BC (2022). Avatars in the metaverse: potential legal issues and remedies. Int Cybersecur Law Rev.

[36] Dahan NA, Al-Razgan M, Al-Laith A, Alsoufi MA, Al-Asaly MS, Alfakih T (2022). Metaverse framework: a case study on E-learning environment (ELEM). Electronics.

[37] Yang F-CO, Lo F-YR, Hsieh JC, Wu W-CV (2020). Facilitating communicative ability of EFL learners via high-immersion virtual reality. J Educational Technol Soc.

[38] Jaynes C, Seales WB, Calvert K, Fei Z, Griffioen J. The metaverse: a networked collection of inexpensive, self-configuring, immersive environments. In: *Proceedings of the workshop on Virtual environments 2003: 2003*; 2003:115–124.

[39] Wei Q, Wu H, Shi F, Wan Y, Ning H. A tutorial on meta-services and services computing in metaverse. IEEE Internet Things. 2023.

[40] Bojic L (2022). Metaverse through the prism of power and addiction: what will happen when the virtual world becomes more attractive than reality?. Eur J Futures Res.

[41] Chen M (2022). Digital affordances and teacher agency in the context of teaching Chinese as a second language during COVID-19. System.

[42] Bahari A (2022). Affordances and challenges of teaching language skills by virtual reality: a systematic review (2010–2020). E-Learning and Digital Media.

[43] Lan Y-J. Does second life improve Mandarin learning by overseas Chinese students? 2014.

[44] Shi A, Wang Y, Ding N (2022). The effect of game–based immersive virtual reality learning environment on learning outcomes: designing an intrinsic integrated educational game for pre–class learning. Interact Learn Environ.

[45] Mantziou O, Papachristos NM, Mikropoulos TA (2018). Learning activities as enactments of learning affordances in MUVEs: a review-based classification. Educ Inform Technol.

[46] Abdullah J, Mohd-Isa WN, Samsudin MA (2019). Virtual reality to improve group work skill and self-directed learning in problem-based learning narratives. Virtual Reality.

[47] Duman S, Çelik Özen D, Duman Ş (2022). Metaverse in paediatric dentistry. Eur Archives Pediatr Dentistry.

[48] Cruz-Lara S, Osswald T, Guinaud J, Bellalem N, Bellalem L, Camal J-P. A chat interface using standards for communication and e-learning in virtual worlds. In: *Enterprise Information Systems: 12th International Conference, ICEIS* 2010, Funchal-Madeira, Portugal, June 8–12, 2010, Revised Selected Papers 12: 2011: Springer; 2011:541–554.

[49] Siyaev A, Jo G-S (2021). Towards aircraft maintenance metaverse using speech interactions with virtual objects in mixed reality. Sensors.

[50] Alfalah SF (2018). Perceptions toward adopting virtual reality as a teaching aid in information technology. Educ Inform Technol.

[51] Villena Taranilla R, Cózar-Gutiérrez R, González-Calero JA, López Cirugeda I (2022). Strolling through a city of the Roman Empire: an analysis of the potential of virtual reality to teach history in primary education. Interact Learn Environ.

[52] Akayoğlu S (2019). Theoretical frameworks used in CALL studies: a systematic review. Teach Engl Technol.

[53] Lantolf JP, Thorne SL. Sociocultural theory and genesis of second language development: Oxford: Oxford University Press, 2006; 2006.

[54] Kramsch C (2009). Cultural perspectives on language learning and teaching. Handb Foreign Lang Communication Learn.

[55] Hennig-Thurau T, Aliman DN, Herting AM, Cziehso GP, Linder M, Kübler RV (2023). Social interactions in the metaverse: Framework, initial evidence, and research roadmap. J Acad Mark Sci.

[56] Ale M, Sturdee M, Rubegni E (2022). A systematic survey on embodied cognition: 11 years of research in child–computer interaction. Int J Child-Comput Interact.

[57] Wilson AD, Golonka S (2013). Embodied cognition is not what you think it is. Front Psychol.

[58] Lakoff G (2012). Explaining embodied cognition results. Top Cogn Sci.

[59] Atkinson D (2010). Extended, embodied cognition and second language acquisition. Appl Linguist.

[60] Dijkstra K, Kaschak MP, Zwaan RA (2007). Body posture facilitates retrieval of autobiographical memories. Cognition.

[61] Siakaluk PD, Pexman PM, Aguilera L, Owen WJ, Sears CR (2008). Evidence for the activation of sensorimotor information during visual word recognition: the body–object interaction effect. Cognition.

[62] Miao F, Kozlenkova IV, Wang H, Xie T, Palmatier RW (2022). An emerging theory of avatar marketing. J Mark.

[63] Gallagher S. Phenomenology and embodied cognition. Routledge Handb Embodied Cognition. 2014:9–18.

[64] Pasfield-Neofitou S, Huang H, Grant S (2015). Lost in second life: virtual embodiment and language learning via multimodal communication. Education Tech Research Dev.

[65] Zheng D, Young MF, Wagner MM, Brewer RA (2009). Negotiation for action: English language learning in game-based virtual worlds. Mod Lang J.

[66] Carruthers G (2013). Toward a cognitive model of the sense of embodiment in a (rubber) hand. J Conscious Stud.

[67] Mejia-Puig L, Chandrasekera T. The virtual body in a design exercise: a conceptual framework for embodied cognition. Int J Technol Des Educ. 2022:1–22.

[68] Oh CS, Bailenson JN, Welch GF (2018). A systematic review of social presence: definition, antecedents, and implications. Front Rob AI.

[69] Schmidt M, Benzing V, Wallman-Jones A, Mavilidi M-F, Lubans DR, Paas F (2019). Embodied learning in the classroom: effects on primary school children’s attention and foreign language vocabulary learning. Psychol Sport Exerc.

[70] Kosmas P, Ioannou A, Zaphiris P (2019). Implementing embodied learning in the classroom: effects on children’s memory and language skills. Educational Media International.

[71] Zhou S, Hiver P, Al-Hoorie AH. Measuring L2 engagement: a review of issues and applications. Student Engagem Lang Classr. 2021:75–98.

[72] Mthethwa-Sommers S. A case study on online learning collaboration and facilitation of a virtual classroom community. Handbook of research on facilitating collaborative learning through digital content and learning technologies. edn.: IGI Global; 2023. pp. 297–310.

[73] Lardier DT Jr, Dickson EL, Hackett JM, Verdezoto CS. A scoping review of existing research between 1990 and 2023: measuring virtual communities of practice across disciplines. J Community Psychol. 2023. 10.1002/jcop.23092 37792285

[74] Ahmady S, Kohan N, Bagherzadeh R, Rakshhani T, Shahabi M (2018). Validity testing of classroom community scale in virtual environment learning: a cross sectional study. Annals of Medicine and Surgery.

[75] Çiğdem H, Öncü S. Learner engagement in the metaverse: a community of inquiry for self-regulated learners. Shaping the future of online learning: education in the metaverse. edn.: IGI Global; 2023. pp. 17–36.

[76] Rockinson-Szapkiw AJ, Wendt J, Whighting M, Nisbet D (2016). The predictive relationship among the community of inquiry framework, perceived learning and online, and graduate students’ course grades in online synchronous and asynchronous courses. Int Rev Res Open Distrib Learn.

[77] Garrison DR. E-learning in the 21st century: a community of inquiry framework for research and practice. Taylor & Francis; 2016.

[78] Garrison DR. Thinking collaboratively: learning in a community of inquiry. Routledge; 2015.

[79] Sun Z, Yang Y (2023). The mediating role of learner empowerment in the relationship between the community of inquiry and online learning outcomes. The Internet and Higher Education.

[80] Kohnke L, Foung D (2023). Promoting positive emotions during the emergency remote teaching of English for Academic purposes: the unexpected role of the Constructionist Approach. Educ Sci.

[81] Pellas N, Kazanidis I, Konstantinou N, Georgiou G (2017). Exploring the educational potential of three-dimensional multi-user virtual worlds for STEM education: a mixed-method systematic literature review. Educ Inform Technol.

[82] Maddrell JA, Morrison GR, Watson GS. Presence and learning in a community of inquiry. Social presence and identity in online learning. edn.: Routledge; 2020. pp. 109–22.

[83] Lee SJ, Huang K (2018). Online interactions and social presence in online learning. J Interact Learn Res.

[84] Hiver P, Al-Hoorie AH, Vitta JP, Wu J. Engagement in language learning: a systematic review of 20 years of research methods and definitions. Lang Teach Res. 2021:13621688211001289.

[85] Li Z, Li J (2022). Using the flipped classroom to promote learner engagement for the sustainable development of language skills: a mixed-methods study. Sustainability.

[86] O’Brien H. Theoretical perspectives on user engagement. Why engagem matters: cross-disciplinary perspect user engagem digit media. 2016:1–26.

[87] O’Brien HL, Cairns P, Hall M (2018). A practical approach to measuring user engagement with the refined user engagement scale (UES) and new UES short form. Int J Hum Comput Stud.

[88] Lalmas M, O’Brien H, Yom-Tov E. Measuring user engagement: Springer Nature; 2022.

[89] Sutcliffe A. Designing for user engagment: aesthetic and attractive user interfaces. Springer Nature; 2022.

[90] Tsai W-HS, Liu Y, Chuan C-H (2021). How chatbots’ social presence communication enhances consumer engagement: the mediating role of parasocial interaction and dialogue. J Res Interact Mark.

[91] Imaoka Y, Amaki Y, Yagi K, Fuller C, Madden A. Metaverse utilization in the EFL classroom-A study. Second life (Register Trademark) as part of an institution’s curriculum. In: *Selected papers presented at MODSIM world 2011 conference and expo: 2012*; 2012.

[92] Grant SJ, Huang H. Learning a second language in second life. Effectively implementing information communication technology in higher education in the Asia-Pacific region. edn.: Nova Science Publishers; 2012. pp. 183–200.

[93] Henderson M, Huang H, Grant S, Henderson L. The impact of Chinese language lessons in a virtual world on university students’ self-efficacy beliefs. Australasian J Educational Technol. 2012;28(3).

[94] Lan Y-J, Kan Y-H, Hsiao IY, Yang SJ, Chang K-E. Designing interaction tasks in second life for Chinese as a foreign language learners: a preliminary exploration. Australasian J Educational Technol. 2013;29(2).

[95] Dominguez-Noriega S, Agudo JE, Ferreira P, Rico M (2011). Language learning resources and developments in the Second Life metaverse. Int J Technol Enhanced Learn.

[96] McVey MH (2008). Observations of expert communicators in immersive virtual worlds: implications for synchronous discussion. Alt-j.

[97] Luo L, Kemp J. Second life: exploring the immersive instructional venue for library and information science education. J Educ Libr Inform Sci. 2008:147–66.

[98] Hayes ER. Situated learning in virtual worlds: The learning ecology of Second Life. 2006.

[99] Baceviciute S, Mottelson A, Terkildsen T, Makransky G. Investigating representation of text and audio in educational VR using learning outcomes and EEG. In: *Proceedings of the 2020 CHI conference on human factors in computing systems: 2020*; 2020:1–13.

[100] Shin D (2022). The actualization of meta affordances: conceptualizing affordance actualization in the metaverse games. Comput Hum Behav.

[101] Cheong D (2010). The effects of practice teaching sessions in second life on the change in pre-service teachers’ teaching efficacy. Comput Educ.

[102] Kongmee I, Strachan R, Pickard A, Montgomery C. Moving between virtual and real worlds: second language learning through massively multiplayer online role playing games (MMORPGs). In: *2011 3rd computer science and electronic engineering conference (CEEC): 2011*: IEEE; 2011:13–18.

[103] Lee K, Kweon S-O, Lee S, Noh H, Lee GG (2014). POSTECH immersive English study (POMY): Dialog-based language learning game. IEICE Trans Inf Syst.

[104] Wang CX, Calandra B, Hibbard ST, McDowell Lefaiver ML (2012). Learning effects of an experimental EFL program in Second Life. Education Tech Research Dev.

[105] Jabbari N, Eslami ZR (2019). Second language learning in the context of massively multiplayer online games: a scoping review. ReCALL.

[106] Shih Y-C, Yang M-T (2008). A collaborative virtual environment for situated language learning using VEC3D. J Educational Technol Soc.

[107] Xu Z, Chen Z, Eutsler L, Geng Z, Kogut A (2020). A scoping review of digital game-based technology on English language learning. Education Tech Research Dev.

[108] Nocchi S. Foreign language teaching and learning in virtual worlds: the construct of affordance. Virtual worlds: concepts applications and future directions. 2018:169–200.

[109] Wang C-p, Lan Y-J, Tseng W-T, Lin Y-TR, Gupta KC-L (2020). On the effects of 3D virtual worlds in language learning–a meta-analysis. Comput Assist Lang Learn.

[110] Peterson M (2011). Towards a research agenda for the use of three-dimensional virtual worlds in language learning. Calico J.

[111] Rogers J, Revesz A. Experimental and quasi-experimental designs. The Routledge handbook of research methods in applied linguistics. edn.: Routledge; 2019. pp. 133–43.

[112] Bhardwaj P (2019). Types of sampling in research. J Prim Care Specialties.

[113] Arbaugh JB, Cleveland-Innes M, Diaz SR, Garrison DR, Ice P, Richardson JC, Swan KP (2008). Developing a community of inquiry instrument: testing a measure of the community of inquiry framework using a multi-institutional sample. The Internet and Higher Education.

[114] Rovai AP (2002). Development of an instrument to measure classroom community. The Internet and Higher Education.

[115] Çelik F. Metaverse based vocabulary class. In.: OSF; 2023.

[116] Makransky G, Mayer RE (2022). Benefits of taking a virtual field trip in immersive virtual reality: evidence for the immersion principle in multimedia learning. Educational Psychol Rev.

[117] Reyes CG (2020). Perception of high school students about using Metaverse in augmented reality learning experiences in mathematics. Pixel-Bit: Media and Education Magazine.

[118] Wu B, Yu X, Gu X (2020). Effectiveness of immersive virtual reality using head-mounted displays on learning performance: a meta‐analysis. Br J Edu Technol.

[119] Chabot S, Drozdal J, Zhou Y, Su H, Braasch J. Language learning in a cognitive and immersive environment using contextualized panoramic imagery. In: *HCI International* 2019-Posters: 21st International Conference, HCII 2019, Orlando, FL, USA, July 26–31, 2019, Proceedings, Part III 21: 2019: Springer; 2019:202–209.

[120] Díaz J, Saldaña C, Avila C (2020). Virtual world as a resource for hybrid education. Int J Emerg Technol Learn (iJET).

[121] Erturk E, Reynolds G-B. The expanding role of immersive media in education. In: *International Conference on E-learning: 2020*; 2020:191–194.

