# Supplementary material for: The effect of metaverse on L2 vocabulary learning, retention, student engagement, presence, and community feeling
Source: BMC Psychol. 2024 Feb 2;12:58. doi: 10.1186/s40359-024-01549-4 (PMC10837982; doi:10.1186/s40359-024-01549-4)
Supplement: Supplementary file 1 — Supplementary Material 1: Vocabulary Learning and Retention Test [file 40359_2024_1549_MOESM1_ESM.pdf]

Supplementary file 1. Vocabulary Learning and Retention Test

Full Name:
Class/No:

1. The number of college students ----- from 214,000 to 127,000.

a) inclined
b) declined
c) justified
d) implemented

2. Parrots are either taken as a pet or they live in the -----.

a) ground
b) air
c) wild
d) urban

3. This forest is ----- visited by humans. It looks like it is untouched.

a) hazardously
b) rarely
c) fatally
d) thoroughly

4. Jack does not like listening to loud music. -----, he usually doesn't join parties.

a) However
b) Nevertheless
c) Because
d) Therefore

5. Tigers ----- deers for a long period of time to feed themselves.

a) track down
b) sit down
c) hand down
d) take in

6. The polar bears are facing a loss of their ----- due to climate change.

a) liberty
b) environment
c) habitat
d) concept

7. The government prevents forestry companies from ----- in this forest.

a) clumsy
b) logging
c) landmark
d) reputation

8. The engineers are working diligently to ----- the new bridge over the river.

a) hamper
b) construct
c) diminish
d) refute

9. The patient was given a new ---- by the doctor to help alleviate their chronic pain.

a) medicine
b) distribution
c) Indication
d) resolution

10. The ---- forest made it hard for the explorers to navigate through the thick underbrush.

a) mortal
b) trivial
c) dense
d) decayed

12. The polar bear's thick ----- protected it from the harsh Arctic winds.

a) cloth
b) fur
c) extinct
d) timidity

13. Due to the destruction of their natural habitat and overhunting, the mountain gorilla has become an ----- species.

a) perspired
b) tempted
c) endangered
d) preferred

14. The ----- of the butterfly was new to the entomologist, and it was carefully studied and documented.

a) adversary
b) proximity
c) species
d) compassion

15. The people usually hunt snow leopards because they are -----.

a) portable
b) indispensable
c) valuable
d) arguable

17. The government applied strict measures to --- air pollution.

a) hype
b) reinforce
c) increase
d) reduce

18. National parks and wildlife reserves aim to ---- natural habitats.

a) exaggerate
b) protect
c) exploit
d) neglect

19. The photographer took a photo and sent it to authorities when someone was illegally ----- a bird in the jungle.

a) shooting
b) caring for
c) rescuing
d) rehabilitating

20. Animal rights activists work tirelessly to raise awareness and fight the animal ----- in various industries.

a) cooperation
b) empathy
c) difference
d) cruelty
