# Supplementary material for: The effect of metaverse on L2 vocabulary learning, retention, student engagement, presence, and community feeling
Source: BMC Psychol. 2024 Feb 2;12:58. doi: 10.1186/s40359-024-01549-4 (PMC10837982; doi:10.1186/s40359-024-01549-4)
Supplement: Supplementary file 2 — Supplementary Material 2: Metaverse-Based Language Teaching Lesson Plan [file 40359_2024_1549_MOESM2_ESM.docx]

| **Class and level: 9th grade, B1**  **Duration:** 40 min | | **Materials:** spatial mobile app or website  **Tools:** mobile phones or tablets  **Metaverse environment:** <https://bit.ly/3GtL3k5> | | |
| --- | --- | --- | --- | --- |
| **Aims & Objectives:** By the end of this lesson the students will be able to…   - talk about endangered animals - learn vocabulary in the context of habitats such as *declining, environment, endangered* - talk about what they would do to preserve endangered animals   **Language Skills**: Four skills integrated with a specific focus on reading and speaking.  **Components Practised** (i.e. Grammar/Vocabulary): Specific focus on vocabulary with a slight focus on form.  **Anticipated Difficulties and Remedies:** (What may happen? What may go wrong? What may not work? What’s your plan B?)The internet connection can be cut off. In this case, the lesson can be proceeded using conventional methods such as drawing, writing on the board, reading aloud etc. Some students may find the technology hard to use or follow the instructions. In this case, it will be okay to pair him/her with a more knowledgeable peer. | | | | |
| **Stages**  **Logical division of the lesson?** | **Procedure**  **What’s the T doing? What are the Ss doing?** | | **Interaction Patterns** | **Duration**  **Allocated time?** |
| **WARM UP** | Ask students to look at the poster on the wall. Explain that endangered species are animals or plants which are in danger of extinction. Ask students if they know any endangered species. Ask for examples. Next, ask if they know of any efforts to protect these animals in the country that they live in or elsewhere? | | Teacher to students. Students to teacher. Class interaction. | *10 min* |
| **PRESENTATION** | Divide the class into two groups: A and B. Tell them that they are going to learn about two different animals. Students that are in group A are going to learn about snow leopards and students in group B are going to learn about mountain gorillas. Ensure that they understand they should only read about their animal. Tell them that there is an interactive dictionary on the side walls where they can look up any words that they don’t know. Have them follow their paths and read and learn about their animals (A students go to the left and B students go to the right of the corridor). | | Students to students Teacher to students | *20 min* |
| **PRACTICE** | Ask students to gather in the hall around the tree. Have them pair with a student from the other group (a student from A matching with a student from B group). Tell them to follow you to the speaking area. Hand out the interview chart to the students. Now, ask them to interview their partners about his or her animal and complete the information in the chart.  Go over the chart with the class and point out the example question. Elicit a question for each of the other items, for instance *habitat* — *Where does the snow leopard live?* etc. Have students ask their partner the questions and complete the chart. Check answers with the class, having students tell you the questions first. | | Students to students Teacher to students Students to teacher | *20 min* |
| **PRODUCTION** | The snow leopard and mountain gorilla are suffering. Both animals are almost extinct. What would you do to raise awareness of their situations? What would you tell people? Discuss with a partner.  Go over the directions with the class, then give them a few moments to think about their answers. Encourage them to write down a few notes with their ideas. Model some examples using *would: I would give a presentation at school. I would tell them about the animals’ situation, I would explain that…*,etc. Write the prompt *I would…* on the sticky note and make it bigger and stick to the wall in the Metaverse. Have students say what comes after *would* (base verb). Assign students to pairs to discuss what they would do. Remind students to ask follow-up questions. Monitor and provide help as necessary. Have students tell the class about the partner’s ideas. | | Class interaction Teacher to students Students to students Students to teacher | *20 min* |
| **HOMEWORK** | Have students create posters with information about one of the animals (or they can research another endangered animal) and send them to you (e.g. via email). The poster should urge people to do something to support protecting animals in danger of extinction. They can either prepare it on paper and take its photo or use digital platforms to create their posters such as Canva and download. Tell them that you will place their posters on the walls of this Metaverse environment and they will have an exhibition in the upcoming days to look at all the posters. | | Teacher to students | *5 min* |

**Supplementary file 2.** Metaverse-Based Language Teaching Lesson Plan
